# The relationship of flavonoid intake during pregnancy with excess body weight and gestational diabetes mellitus

**DOI:** 10.20945/2359-3997000000143

**Published:** 2019-05-25

**Authors:** Mariana de Andrade Balbi, Lívia Castro Crivellenti, Daniela Cristina Candelas Zuccolotto, Laércio Joel Franco, Daniela Saes Sartorelli

**Affiliations:** 1 Universidade de São Paulo Universidade de São Paulo Faculdade de Medicina de Ribeirão Preto (FMRP) Programa de Pós-Graduação em Saúde Pública Ribeirão Preto SP Brasil Programa de Pós-Graduação em Saúde Pública, Faculdade de Medicina de Ribeirão Preto (FMRP), Universidade de São Paulo (USP), Ribeirão Preto, SP, Brasil; 2 Universidade de São Paulo Universidade de São Paulo Faculdade de Medicina de Ribeirão Preto (FMRP) Departamento de Medicina Social Ribeirão Preto SP Brasil Departamento de Medicina Social, Faculdade de Medicina de Ribeirão Preto (FMRP), Universidade de São Paulo (USP), Ribeirão Preto, SP, Brasil

**Keywords:** Pregnancy, flavonoids, gestational diabetes mellitus, overweight, obesity

## Abstract

**Objective:**

To investigate the relationship of flavonoid intake during pregnancy with maternal excessive body weight and gestational diabetes mellitus (GDM).

**Subjects and methods:**

A cross-sectional study was conducted among 785 adult women in singleton pregnancies, and data were collected at the time of the oral glucose tolerance test. For the body mass index (BMI) classification according to the gestational age, the criteria of Atalah was used, and the diagnosis of GDM was based on the World Health Organization of 2014. Two 24-hour dietary recalls were obtained, and the usual intake was determined by the Multiple Source Method. Adjusted multinomial logistic regression was used to investigate the relationship of the flavonoids with overweight and obesity, and adjusted non-conditional logistic regression for the relationship of the flavonoids with GDM.

**Results:**

The mean (SD) age of the women was 28 (5) years, 32.1% were overweight, 24.6% were obese and 17.7% were diagnosed with GDM. The median (P25, P75) of total flavonoid intake was 50 (31,75) mg/day. Considering the eutrophic women as the reference, the pregnant women with a higher total flavonoid intake [OR 0.62 (95% CI 0.38; 0.96)] and anthocyanidin intake [OR 0.62 (95% CI 0.40; 0.99)] were less likely to be obese when compared to the women with lower intakes. No association of the flavonoids intake with overweight or GDM was found.

**Conclusion:**

A very low intake of flavonoids was observed. The data suggest that the intake of foods naturally rich in total flavonoids and anthocyanidin has a beneficial role regarding obesity among pregnant women.

## INTRODUCTION

Maternal excess weight is a relevant risk factor for deleterious maternal and fetal health outcomes in the short- and long-term (1,2) and is the main determinant of gestational diabetes mellitus (GDM) (3). Globally, it is estimated that approximately 16% of pregnant women are affected by hyperglycemia (3). Therefore, investigations into factors with potential protective effects for the disease are of paramount importance.

Evidence suggests that diets composed of plant-rich foods high in bioactive compounds, such as flavonoids, have a protective role for type 2 diabetes mellitus (DM) (4) and obesity (5). Flavonoids belong to the group of polyphenols and are classified into six subclasses: flavan-3-ols, anthocyanidins, flavonols, flavanones, flavones and isoflavones (6). In vitro and in vivo studies have demonstrated that flavonoids have anti-inflammatory and antioxidant properties (7), with a high potential for modulation of the intestinal microbiota (8) and the inflammatory response induced by excess weight (9). It has been suggested that flavonoids can act in glycemic homeostasis through several mechanisms: inhibiting glucose absorption; stimulating insulin secretion by pancreatic β cells; favoring the modulation of glucose release by the liver; and acting on the improvement of the action of insulin receptors and absorption of glucose by the tissues (6).

Epidemiological studies indicate an inverse relationship between flavonoid intake and adiposity indicators (10–13). Hughes and cols. (14) observed an increase in the body mass index (BMI) over the years among women with lower intakes of flavonols, flavones and catechins. In a study conducted with 2,734 adult twins, it was found that a higher habitual intake of flavonoids, including anthocyanins, flavan-3-ols, flavonols and proanthocyanidins, was associated with a lower percentage of body fat mass, regardless of confounding factors (12).

Epidemiological studies conducted in the United States and Europe suggest a protective effect of dietary flavonoids for the development of DM, regardless of confounding factors (4,15–17). European cohort data suggest a lower incidence of DM among individuals with higher habitual flavonoid intake (16). In the United States, cohort findings corroborate this hypothesis (17). In a dose-response analysis of a meta-analysis of prospective studies, Liu and cols. (4) concluded that the intake of 500 mg/day of flavonoids was associated with a 5% reduction in the risk of DM.

During pregnancy there is a profound transformation in the intestinal microbiota of women that may interfere with the absorption and metabolism of flavonoids (18). Although investigations in vitro and in animal models suggest a potential protective effect of flavonoids for GDM (6), no epidemiological studies were found that investigated the relationship of the intake of these compounds in pregnancy with excess weight and GDM. The hypothesis of the present study was an inverse relationship between the intake of flavonoids with excess body weight and GDM. The aim of the present study was to investigate the relationship of intake of total flavonoids and subclasses during gestation with maternal excess weight and GDM.

## SUBJECTS AND METHODS

### Study design and population

A cross-sectional study conducted with 785 women, in singleton pregnancies, receiving prenatal care in the Public Health System of Ribeirão Preto, São Paulo state, Brazil (19). The recruitment of women was carried out by trained nutritionists, when the oral glucose tolerance test (OGTT) was performed, and all the pregnant women who attended these laboratories to carry out the OGTT, between 2011 and 2012, were invited to participate in the study. At the time of the assessment, neither the women nor the interviewer knew about the results of the OGTT.

The study inclusion criteria were: age 20 years or over and pre-gestational body mass index 20 kg/m^2^ or over. The exclusion criteria were: report of pre-pregnancy diabetes; pregnant women with GDM screening prior to the 24^th^ gestational week; twin pregnancy, report of use of glycemia altering drugs (such as glucocorticoids); and reports of diseases that alter habitual food consumption. Women with fasting blood glucose ≥126 mg/dL or ≥200 mg/dL two hours after glucose overload were considered to have prior DM and were subsequently excluded from the study.

A total of 1,446 women were contacted, however, 19 women refused to participate in the study, 639 were excluded according to the established criteria and three pregnant women presented incomplete data, giving a sample total of 785 women (19). Assuming a prevalence of 20% of GDM among women attended in the Brazilian Nation Health System, a sample size of 512 individuals was necessary, with a margin of error of 5% (20). For the calculation of the gestational week (GW), the date of the last menstruation recorded on the medical card of the pregnant woman was used. The gestational age at the time of the interview ranged from 24 to 39 weeks (70% of the women were between the 24^th^ and 28^th^ GW, 21.5% between the 29^th^ and 32^nd^ GW and 8.5% ≥33 GWs).

The study was approved by the Research Ethics Committee of the School Health Center of the Ribeirão Preto Medical School (075/2015-CEP/CSE-FMRPUSP) of the University of São Paulo. The pregnant women agreed to participate in the study by signing the informed consent form.

### Classification of BMI according to gestational age

Data regarding weight (kg) and height (m) were obtained by trained nutritionists at the time of the OGTT using a digital scale (Tanita, model HS 302) and a portable stadiometer (Sanny, model ES 2040), respectively. The current BMI was calculated, with the criteria proposed by Atalah and cols. used for the classification of BMI according to gestational age (21). The criterion proposed by Atalah and cols. (21) was developed using data of Chilean women, and presents limitations to be used in Brazil. However, this criterion was adopted by the Brazilian Ministry of Health to evaluate pregnant women in the country (22).

### Diagnosis of gestational diabetes mellitus

Blood samples were obtained after 12 hours of fasting, followed by the ingestion of a 75g glucose overload by the participants. Determination of fasting glycemia, one and two hours after overload, was performed using the glucose oxidase test. The diagnosis of GDM was performed according to the criteria of the World Health Organization of 2014 (23), considering GDM if at least one of the glycemia values were altered in the women, at any stage of the gestation: fasting glycemia between 92 and 125 mg/dl; one hour after glucose overload ≥180 mg/dl; or glucose two hours after glucose overload between 153 and 199 mg/dl.

### Estimation of flavonoids from the usual diet

The food intake assessment was performed through two 24-hour dietary recalls on non-consecutive days, adopting the “multiple-pass” technique in three stages (24). The first 24-hour dietary recall was obtained at the time of the interview and the second through telephone contact, with at least seven days between replications, regardless the day of the week. The energy intake was estimated using the NutWin^®^ software (Nutrition Support Program, Version 1.5, São Paulo, Brazil, 2002), adopting the database of the Brazilian Food Composition Table. For the estimation of total flavonoids and subclasses of most foods, the Brazilian Food Composition Table (TBCA-USP) (25,26) was used. For the estimative of flavonoids of foods without data on the TBCA, the USDA Database for the Flavonoid Content of Selected Foods was used (27). In the present study, the subclasses of flavonoids explored were: flavonols, flavones, flavanones, flavan-3-ols and anthocyanidins. Isoflavones were not quantified due to low intake of food sources among the study participants.

To estimate the usual intake of flavonoids and subclasses in the diet the Multiple Source Method (MSM) was employed. The MSM is a statistical modeling program developed by the European Prospective Investigation into Cancer and Nutrition to estimate the usual diet (web-based https://msm.dife.de/) (28). The method estimates the usual intake of nutrients through three steps: 1. Estimation of probability of intake on a random day; 2. Estimation of usual intake on days of consumption, corrected for variability; 3. The product of the probability of intake (step 1) divided by the usual intake on the day of consumption (step 2) results in the usual intake. The MSM dispenses a high number of 24-hour dietary recalls (or food records) replications (29). In the present study, the response rate for the second 24-hour dietary recall was 62%, considered adequate for analysis using the MSM (30). In a previous study it was observed that the MSM was an adequate method to estimate the usual food intake during pregnancy (31).

### Covariables

Information on age (years), education (years of study), smoking (never smoked, ex-smoker and current smoker), practice of physical activity (minutes/week of walking and physical exercise), parity, family history of diabetes and history GDM in previous pregnancies were obtained through a structured questionnaire at the time of the interview during the OGTT. Pre-gestational weight was calculated based on data recorded on the obstetric monitoring card of the pregnant woman.

### Statistical analysis

The study population was characterized using the mean (SD) and median (P25, P75) values for the continuous descriptive variables, while the categorical variables were expressed as frequencies. The dietary intake data were log transformed, and T Student test and ANOVA with post-hoc of Bonferroni were used to investigate differences in the estimated flavonoids of pregnant women according to glycemic homeostasis and the BMI categories, respectively.

The MSM was used to estimate the usual intake of total flavonoids and subclasses. For inclusion in the models, the estimate of flavonoids was transformed into its natural logarithm and adjusted for total energy through the residual method. The data were later categorized into intake tertiles.

Multinomial logistic regression models were used to evaluate the relationship between total flavonoids and subclasses (in tertiles) and the BMI categories according to gestational week, considering the eutrophic women as the reference. In the analysis, 31 women with low weight according to the gestational week were excluded. The following variables were considered in the adjusted models: age (years), education (years of study), gestational week at the time of interview, smoking (never smoked, ex-smoker, current smoker), practice of physical activity (minutes/week), parity (number of children) and total energy of the diet (log-transformation, kcal/day).

Adjusted logistic regression models were used to estimate the relationship of dietary content of flavonoids and subclasses with GDM, adjusted for age (years), education (years of study), gestational week at the time of the interview, smoking (never smoked, ex-smoker, current smoker), practice of physical activity (minutes/week of walking and exercise), parity (number of children) pre-gestational BMI (kg/m^2^), current BMI (kg/m^2^), family history of diabetes (yes/no), history of GDM (yes/no) and total dietary energy (log-transformation, kcal/day).

The significance level was set at *p*<0.05 and all statistical analyses were performed using the SPSS software (Version 24.0, SPSS Inc. Woking, Surrey, UK).

## RESULTS

Among the 785 study participants, 17.7% were diagnosed with GDM, and 56.7% had excessive body weight. The age of the women ranged from 20 to 45 years and the education level between zero and 15 years of study (Table 1).

**Table 1:** Sociodemographic, anthropometric and lifestyle characteristics of the pregnant women. Ribeirão Preto, SP, 2011-2012. *n* = 785

Maternal characteristics		Frequency (n%)
Current GDM^a^		139 (17.7)
History of GDM in previous pregnancies		34 (4.3)
Family history of diabetes		205 (26.1)
BMI classification (kg/m^2^)^b^		
	Low weight	31 (3.9)
	Eutrophic	309 (39.4)
	Overweight	252 (32.1)
	Obese	193 (24.6)
Smoking		
	Never smoked	624 (79.5)
	Ex smoker	90 (11.5)
	Current smoker	71 (9.0)
		**Mean (SD)**
Age (years)		27.6 (5.5)
Gestational age at time of interview		27.7 (3.2)
Education (years of study)		9.2 (2.7)
Pre-gestational BMI (kg/m^2^)		25.9 (5.0)
Parity		1.2 (1.2)
		**Median (P25; P75)**
Physical activity^c^		40 (0; 140)

a According to World Health Organization criteria. (22)

b Classification of current BMI according to gestational week. (21)

c Minutes per week of walking and physical exercise.

It was observed that the drinks and foods that contributed the most to the intake of total flavonoids of the pregnant women's diet were: mate tea, beans, oranges, chocolate powder and orange juice, as presented in Table 2.

**Table 2:** Contribution of foods and beverages to the intake of total flavonoids and subclasses of the pregnant women. Ribeirão Preto, SP, 2011-2012. *n* = 785

Food and beverages	Contribution (%)
**Total flavonoids**	
Mate tea	45.0
Beans (brown)	11.0
Oranges	8.0
Chocolate powder	7.0
Orange juice	6.0
Others	23.0
**Anthocyanidins**	
Açaí	34.0
Grapes (purple)	33.0
Strawberries	8.0
Plums	5.0
Blackberries	5.0
Others	15.0
**Flavan-3-ols**	
Chocolate powder	49.0
Apples	19.0
Bananas	16.0
Chocolate	6.0
Mangos	2.0
Others	8.0
**Flavanones**	
Oranges	49.0
Orange juice	39.0
Tangerines	10.0
Lemons	1.0
Wine (red)	0.1
Others	0.9
**Flavones**	
Spring onions	56.0
Chicory	15.0
Watermelon	7.0
Pumpkin	6.0
Grapes (purple)	4.0
Others	12.0
**Flavonols**	
Mate tea	69.0
Beans (brown)	16.0
Arugula	3.0
Apples	3.0
Lettuce	1.0
Others	8.0

The median (P25, P75) of total flavonoids of the pregnant women was 50 (31, 75) mg. Women with adequate BMI reported a higher intake of anthocyanidin and flavan-3-ol, when compared with those classified with obesity. Moreover, pregnant women with adequate BMI reported a higher intake of flavanones, when compared with those classified with overweight. However, no significant difference was observed according to the glycemic homeostasis (Table 3).

**Table 3:** Median (P25; P75) intake of total flavonoids and subclasses according to the glycemic homeostasis and BMI categories of the pregnant women. Ribeirão Preto, SP, 2011-2012

	Glycemic homeostasis^a^ (*n* = 785)			BMI classification^b^(*n* = 754)		
	All women (*n* = 785)	Normal (*n* = 646)	GDM (*n* = 139)	Adequate (*n* = 309)	Overweight (*n* = 252)	Obesity (*n* = 193)
Total Flavonoids	50.0 (31.0; 75.0)	51.0 (31.9; 75.1)	47.3 (29.7; 77.3)	57.2 (33.4; 80.9)	49.0 (32.2; 73.3)	45.4 (28.7; 65.0)
Anthocyanidins	1.9 (0.8; 4.7)	1.9 (0.8; 4.6)	1.9 (0.7; 5.7)	2.2 (0.9; 6.2)	1.8 (0.8; 4.7)	1.7 (0.6; 3.9)^c^,^d^
Flavan-3-ols	7.8 (2.8; 15.2)	7.9 (2.8; 15.2)	7.3 (2.8; 14.4)	8.5 (3.0; 16.0)	8.0 (2.3; 15.9)	6.7 (2.4; 13.0)^c^,^d^
Flavanones	2.3 (1.6; 4.2)	2.3 (1.6; 4.1)	2.2 (1.5; 15.7)	2.4 (1.6; 21.6)	2.1 (1.5; 3.2)	2.4 (1.8; 4.1)^c^,^e^
Flavones	0.3 (0.2; 0.6)	0.3 (0.2; 0.6)	0.4 (0.2; 0.7)	0.3 (0.2; 0.6)	0.3 (0.2; 0.5)	0.3 (0.2; 0.5)
Flavonols	24.8 (18.4; 31.9)	24.9 (18.7; 31.7)	24.4 (16.5; 32.8)	25.6 (19.2; 33.1)	24.6 (19.0; 31.1)	24.1 (16.6; 31.5)

a According to the WHO criteria. (22)

b According to the criteria of Atalah. (21) A total of 31 women with low weight according to gestational age were excluded in this analysis.

c *p* < 0.05; according to ANOVA. Variables were log transformed prior to the analysis.

d Mean difference between women with adequate BMI, when compared with women classified with obesity.

e Mean difference between women with adequate BMI, when compared with women classified with overweight.

In adjusted multinomial logistic regression models, it was observed that the women classified in the third tertile of the total flavonoid and anthocyanidin estimate had a lower chance of obesity when compared to the women in the first tertile. A direct association between the second tertile of flavanones and obesity was observed, however this association was not verified for the third tertile (Table 4).

**Table 4:** Association of dietary flavonoid intake during pregnancy with excess weight and obesity. Ribeirão Preto, SP, 2011-2012. *n* = 754^a^

			1^st^ tertile	2^nd^ tertile		3^rd^ tertile	
				Odds Ratio^b^	95%CI	Odds Ratio^b^	95%CI
Total Flavonoids							
	Median (mg/day)		26.29		50.44		89.29
		Overweight	1.00 (Ref.)	1.21	0.80; 1.85	0.75	0.49; 1.13
		Obesity	Ref.	1.27	0.81; 1.99	0.61	0.38; 0.96
Anthocyanidins							
	Median (mg/day)		0.58		1.93		7.06
		Overweight	Ref.	0.85	0.56; 1.30	0.75	0.50; 1.31
		Obesity	Ref.	1.01	0.65; 1.58	0.62	0.40; 0.99
Flavan-3-ols			1.00				
	Median (mg/day)		2.41		7.82		17.99
		Overweight	Ref.	1.23	0.81; 1.88	1.05	0.69; 1.60
		Obesity	Ref.	0.89	0.57; 1.40	0.72	0.46; 1.14
Flavanones			Ref.				
	Median (mg/day)		1.39		2.25		24.36
		Overweight	Ref.	1.19	0.79; 1.79	0.67	0.44; 1.02
		Obesity	Ref.	2.17	1.36; 3.47	1.24	0.77; 1.98
Flavones			1.00				
	Median (mg/day)		0.15		0.32		0.75
		Overweight	1.00	1.19	0.79; 1.79	0.88	0.58; 1.32
		Obesity	1.00	1.10	0.71; 1.73	0.84	0.54; 1.33
Flavonols			1.00				
	Median (mg/day)		15.82		24.82		35.25
		Overweight	1.00	1.21	0.80; 1.83	0.85	0.56; 1.31
		Obesity	1.00	0.65	0.41; 1.03	0.75	0.49; 1.17

a 31 women with low weight according to the gestational week were excluded in this analysis.

b Multinomial logistic regression models, considering the eutrophic women as the reference, adjusted for: age (years), gestational week at the time of interview, education (years of study), smoking (never smoked, ex-smoker and current smoker), physical activity (minutes/week of walking, running or physical exercise), number of children and total energy of the diet (log-transformation, kcal/day).

Table 5 presents the results of the relationship of the estimate of total flavonoids from the usual diet and their subclasses with GDM. In adjusted logistic regression models, no association was observed.

**Table 5:** The relationship between dietary flavonoid intake during pregnancy and gestational diabetes mellitus. Ribeirão Preto, SP, 2011-2012. *n* = 785

		1^st^ tertile	2^nd^ tertile	3^rd^ tertile	*p*
Total flavonoids					
	Median (mg/day)	26.33	50.33	88.32	
	Odds Ratio (95%CI)^a^	1.00 (Ref.)	0.75 (0.46; 1.21)	1.11 (0.69; 1.78)	0.68
Anthocyanidins					
	Median (mg/day)	0.58	1.91	7.06	
	Odds Ratio (95%CI)^a^	Ref.	1.13 (0.70; 1.83)	1.17 (0.72; 1.89)	0.52
Flavan-3-ols					
	Median (mg/day)	2.41	7.83	17.99	
	Odds Ratio (95%CI)^a^	Ref.	1.07 (0.67; 1.71)	0.84 (0.51; 1.37)	0.49
Flavanones					
	Median (mg/day)	1.38	2.24	24.31	
	Odds Ratio (95%CI)^a^	Ref.	0.78 (0.48; 1.28)	1.11 (0.69; 1.78)	0.66
Flavones					
	Median (mg/day)	0.15	0.32	0.75	
	Odds Ratio (95%CI)^a^	Ref.	1.02 (0.62; 1.69)	1.56 (0.97-2.52)	0.06
Flavonols					
	Median (mg/day)	15.94	24.85	35.30	
	Odds Ratio (95%CI)^a^	Ref.	0.68 (0.68; 1.10)	0.97 (0.61; 1.54)	0.89

a Logistic regression models adjusted for: age (years), education (years of study), gestational week at the time of interview, history of GDM (yes/no), family history of DM (yes/no), smoking (never smoked, ex-smoker and current smoker), practice of physical activity (minutes/week of walking, running or exercise), number of children, pre-gestational BMI (kg/m^2^), current BMI (kg/m^2^) and total dietary energy (log-transformation, kcal/day).

## DISCUSSION

In the present study, an inverse association of the estimate of the usual intake of total flavonoids and anthocyanidins with obesity during gestation was observed, regardless of confounding factors. The women with higher intakes of total flavonoids and anthocyanidins had a 39% and 38% lower chance, respectively, of being classified as having obesity when compared to the women with lower intakes. However, there was no association between dietary flavonoids and overweight or GDM, probably due to the very low intake of flavonoids observed in the study population. No previous epidemiological studies were found that investigated the relationship between dietary flavonoids during pregnancy and maternal excess weight or GDM.

A direct association between the second tertile of flavanones and obesity was observed. To our knowledge, no published data show a detrimental effect of flavanones on weight control, and this relationship probably results from chance.

The median estimate of total flavonoids in the usual diet of the present study (50 mg) was similar to that previously reported among Brazilian adults (55 mg) (32), however, it was much lower than that reported among European adults (246 mg) (16). In the Brazilian study conducted in the city of São Paulo, SP, the main sources of dietary flavonoids were citrus fruits, juices and beans (32); in the present study, the main sources were mate tea, beans and oranges. Worldwide, the main sources of flavonoids are the fruits, vegetables, and wine. In the present study, the median (P25, P75) intake of fruits and vegetables were 67 (38, 136) g/day and 78 (52, 112) g/day, respectively, and the red wine was rarely consumed (data not shown), which could partly explain the very low intake of flavonoids observed.

Given the effect of flavonoids on the maintenance of pancreatic β-cell function and insulin sensitivity [6], one of the hypotheses of the present study was an inverse relationship between their intake and GDM, which was not confirmed. It should be noted that many of the epidemiological studies that have verified a protective effect of flavonoids for DM were conducted in Europe (15,16), where the habitual intake of these compounds is much higher than that found among the pregnant women included in the present study, which could partially explain the controversial findings. In the study by Tresserra-Rimbau and cols. (15), it was verified that the protective effect of flavonoids in relation to DM was observed among individuals with a flavonoid intake higher than 425 mg/day when compared to those who reported a median intake of 291 mg/day. In a secondary analysis of data from the Nurses Health Study, women with a median intake of 718 mg/day of total flavonoids had a lower risk of DM when compared to women with a median intake of 105 mg/day (17). In a meta-analysis of prospective studies it was concluded that intake of 500 mg/day of flavonoids would be necessary for a 5% reduction in DM risk, ten times higher than the median intake found in the study population (4). However, the flavonoids intake by the pregnant women was sufficient to detect an inverse association with obesity.

The inverse association between anthocyanidins in the diet of pregnant women and obesity verified in the present study corroborates the findings of recent epidemiological studies (11,13,17). In secondary analyses of data from the National Health and Nutrition Examination Survey (NHANES 2007-2010 and 2007-2012) in the United States, it was observed that adult individuals with higher intake of anthocyanidins had a 14% lower chance of having high BMI and waist circumference when compared to individuals with lower intakes (10), with an inverse association with BMI also found (33). In the analysis of data from the Harvard cohort studies, there was a greater chance of weight maintenance in individuals with high anthocyanidin intake when compared to participants with low intake over a four-year monitoring period (13).

Experimental studies suggest that the intake of anthocyanidins may contribute to weight control through its modulatory effect on neuropeptide Y and the Y-butyric amino acid receptor in the hypothalamus, thus acting on appetite control (34). However, in the present study, the inverse association between flavonoids and obesity was independent of the total energy intake, as found in previous epidemiological studies (10–12). It has also been suggested that flavonoids can act to reduce visceral fat accumulation and hyperglycemia by inhibiting pancreatic lipase activity and by reducing intestinal lipid absorption (35). Furthermore, recent studies suggest that anthocyanidin exerts a modulating effect on the intestinal microbiota, contributing to the control of obesity [8].

The present study pioneered the investigation of the relationship between flavonoid intake during pregnancy and maternal excess weight and GDM using an epidemiological design. Another strength of the study is that the data were collected by trained nutritionists. The estimation of the flavonoid intake was based on two 24-hour dietary recalls and the estimate of usual intake was performed using the MSM. For the estimation of the flavonoid content of most foods the data determined in Brazilian studies was used. The cross-sectional design constituted the main limitation of the study, however, at the time of the interview neither the women nor the interviewers knew the result of the OGTT, reducing the chance of information bias. Since there is no data on food content of other phenolic compounds available in Brazil, other classes of polyphenols were not determined. The main sources of flavonoids are also rich in other components with antioxidant capacity, which were not estimated in the present study. No data on biomarkers of flavonoid intake was available. In addition, under-reporting of food consumption may have occurred. In addition, due to the lack of information about weight gain in the first trimester of gestation, it was not possible to investigate the relationship between flavonoid intake and gestational weight gain. The adoption of the criterion proposed by Atalah and cols. (21) for the classification of BMI according to gestational age in Brazilian pregnant women is controversial (36), but we do not know another classification available for this population. In addition, this criterion is recommended by the Ministry of Health for the evaluation of pregnant women in the country (22). Due to some evidence for a negative effect of polyphenols in fetal constriction of the ductus arteriosus, intervention studies promoting the intake of flavonoids conducted with pregnant women are not recommended (37).

In conclusion, a low intake of flavonoids was found among the pregnant women, however, in sufficient quantity to detect an inverse association of total flavonoids and anthocyanidin with obesity. There was no association between dietary flavonoids and GDM. However, observational studies conducted among populations with a higher usual intake of flavonoids are needed to elucidate their relationship with GDM.
